# Pro-Apoptotic and Anti-Cancer Properties of Diosgenin: A Comprehensive and Critical Review

**DOI:** 10.3390/nu10050645

**Published:** 2018-05-19

**Authors:** Gautam Sethi, Muthu K. Shanmugam, Sudha Warrier, Myriam Merarchi, Frank Arfuso, Alan Prem Kumar, Anupam Bishayee

**Affiliations:** 1 Department for Management of Science and Technology Development, Ton Duc Thang University, Ho Chi Minh City 700000, Vietnam; 2 Faculty of Pharmacy, Ton Duc Thang University, Ho Chi Minh City 700000, Vietnam; 3 Department of Pharmacology, Yong Loo Lin School of Medicine, National University of Singapore, Singapore 117600, Singapore; phcsmk@nus.edu.sg (M.K.S.); myriammerarchi@hotmail.fr (M.M.); phcapk@nus.edu.sg (A.P.K.); 4 Division of Cancer Stem Cells and Cardiovascular Regeneration, Manipal Institute of Regenerative Medicine, Manipal University, Bangalore 560065, India; sudha.warrier@manipal.edu; 5 Stem Cell and Cancer Biology Laboratory, School of Biomedical Sciences, Curtin Health Innovation Research Institute, Curtin University, Perth, WA 6102, Australia; frank.arfuso@curtin.edu.au; 6 Department of Pharmaceutical Sciences, College of Pharmacy, Larkin University, 18301 N. Miami Avenue, Miami, FL 33169, USA

**Keywords:** diosgenin, steroidal sapogenins, anti-cancer, apoptosis, oncogenic, metastasis

## Abstract

Novel and alternative options are being adopted to combat the initiation and progression of human cancers. One of the approaches is the use of molecules isolated from traditional medicinal herbs, edible dietary plants and seeds that play a pivotal role in the prevention/treatment of cancer, either alone or in combination with existing chemotherapeutic agents. Compounds that modulate these oncogenic processes are potential candidates for cancer therapy and may eventually make it to clinical applications. Diosgenin is a naturally occurring steroidal sapogenin and is one of the major bioactive compounds found in dietary fenugreek (*Trigonella foenum-graecum*) seeds. In addition to being a lactation aid, diosgenin has been shown to be hypocholesterolemic, gastro- and hepato-protective, anti-oxidant, anti-inflammatory, anti-diabetic, and anti-cancer. Diosgenin has a unique structural similarity to estrogen. Several preclinical studies have reported on the pro-apoptotic and anti-cancer properties of diosgenin against a variety of cancers, both in in vitro and in vivo. Diosgenin has also been reported to reverse multi-drug resistance in cancer cells and sensitize cancer cells to standard chemotherapy. Remarkably, diosgenin has also been reported to be used by pharmaceutical companies to synthesize steroidal drugs. Several novel diosgenin analogs and nano-formulations have been synthesized with improved anti-cancer efficacy and pharmacokinetic profile. In this review we discuss in detail the multifaceted anti-cancer properties of diosgenin that have found application in pharmaceutical, functional food, and cosmetic industries; and the various intracellular molecular targets modulated by diosgenin that abrogate the oncogenic process.

## 1. Introduction

Cancer is a complex and heterogeneous disease that afflicts men and women worldwide; it is expected to increase due to human lifestyle changes and a rapidly aging population [1]. Hanahan and Weinberg’s proposed hallmarks of cancer are widely accepted towards understanding the biology of cancer cells, through a multi-stage and progressive process [2,3,4,5,6]. These hallmarks include sustained proliferation of cells, constitutive activation of pro-survival transcription factors, deregulated cellular functions, evading cell death signals and growth suppressors, an increased pro-inflammatory tumor microenvironment, camouflaging against immune cell destruction, promoting angiogenesis, activating cell movement from the primary site and metastasis, enabling replicative immortality, and finally, severe genome instability [2,3,5,6,7,8,9,10]. In addition, several deregulated cellular signaling networks underlying the above hallmarks have been extensively investigated in pre-clinical and clinical drug development [3,4,11,12,13,14,15,16,17,18]. In spite of detailed information of these semi-synthetic and synthetic anti-cancer agents, they only provide limited therapeutic advantages to patients due to highly toxic unwanted side effects and the development of chemoresistance [19,20,21]. Emerging approaches should include the identification of novel drug targets that are very effective in inhibiting the growth of cancer cells, while exhibiting fewer adverse effects. Natural products are a good source of compounds with novel chemical structures that are effective and less toxic [18,22,23,24,25,26,27]. High throughput technologies should be exploited for the screening of a large library of compounds for their anti-cancer activities [28,29,30]. The mainstream of United States Food and Drug Administration (US FDA)-approved compounds divulge that natural products and their derivatives occupy one-third of all novel drugs [22,23,24,31]. These natural compounds, in general, show multi-targeted effects and can modulate several oncogenic transcription factors that block the tumor microenvironment targets that usually sustain tumor growth [22,27,32]. These compounds can also be classified as cytotoxic or cytostatic compounds [30]. Therefore, many secondary metabolites and pure molecules isolated from herbs, spices, dietary fruits and vegetables, and even marine sources have been explored [33,34,35]. 

**Figure 1:**
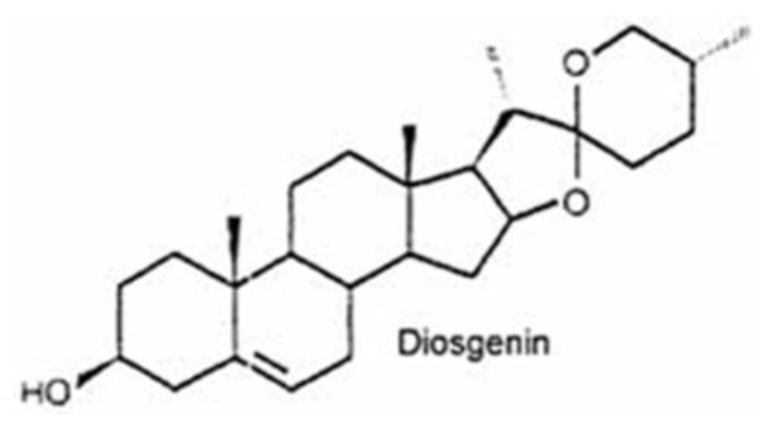
Chemical structure of diosgenin.

**Figure 2:**
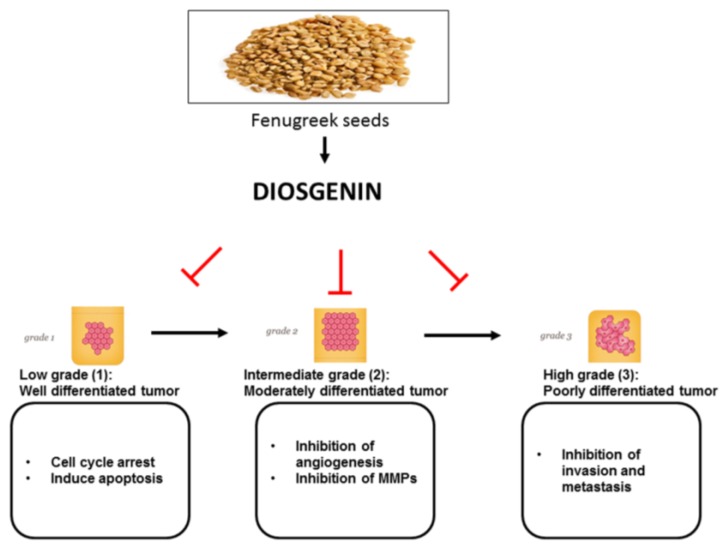
Tumor stage-specific inhibition of molecular targets by diosgenin.

Several novel bioactive molecules have been found in various edible cereals, pulses, roots, and in several parts of medicinal plants. Fenugreek (*Trigonella foenum-graecum*) belongs to the family Leguminosae and is considered a traditional medicinal herb that is commonly used in India, China, Thailand, and South-East Asian countries; it is also cultivated in the Mediterranean region and Northern Africa [36,37,38,39,40,41]. Fenugreek seeds, shoots and leaves are used in Indian curry preparation as a condiment. Bibliometric data indicates that fenugreek extract has several pharmacological properties, such as being hypocholesterolemic, a lactation aid, antibacterial, a gastric stimulant, anti-anorexic, antidiabetic, galactogogic, hepatoprotective, and anti-cancer both in in vitro and in vivo studies [40,41,42,43]. Fenugreek seed extract contains several bioactive molecules in various classes of compounds, such as saponins, flavonoids, coumarins, and alkaloids that target several molecules involved in inflammation and cancer cell proliferation, invasion, migration, angiogenesis, and metastasis [40]. Sapogenins are a class of compounds that widely occur in natural products in their glycoside form and promote general healthy living. Among these compounds, steroidal sapogenins (otherwise known as spirostans) are the most potent bioactive compounds isolated from natural product sources [44,45]. Steroidal sapogenins exhibit ubiquitous pharmacological properties and the majority of them demonstrate anti-cancer activity in vitro and in pre-clinical animal models. Several clinical trials have been conducted with fenugreek seed extract, they are either completed or ongoing; however, diosgenin anti-cancer trials are yet to start [36,41]. Diosgenin is the most abundant steroidal sapogenin in fenugreek seeds. Fujii and Matsukawa isolated and identified diosgenin from *Dioscorea tokoro* Makino in 1935 [41,46] (Figure 1). Diosgenin is a phytosteroidal saponin and a major bioactive compound found in the seeds of *T. foenum-graecum*, commonly known as fenugreek, and in the roots of wild yam (*Dioscorea villosa*) [36,40,41,47,48]. Interestingly, diosgenin is also found in high levels in numerous plant species including *Costus speciosus*, *Smilax menispermoidea*, species of *Paris*, *Aletris* and *Trillum*, and in species of *Dioscorea* [41,49,50]. The steroidal saponin, diosgenin, is biosynthesized from cholesterol. Cholesterol is formed from lanosterol and catalysed by the cytochrome P450 system. Several other routes of synthesis have been identified, such as from squalene-2,3-oxide in two ways: From cycloartenol through the formation of sitosterol [51] and from lanosterol via cholesterol [52]. Several studies have demonstrated the diverse biological activities of diosgenin, such as hypolipidemic, anti-inflammatory, anti-proliferative, hypoglycemic activity, and as a potent anti-oxidant [44]. In addition, diosgenin inhibited cancer cell proliferation and induced apoptosis in a variety of cancer cell lines including colorectal, hepatocellular, breast, osteosarcoma, and leukemia [53,54,55]. The primary mechanism of action of diosgenin is through the modulation of multiple cell signaling pathways that play prominent roles in cell-cycle regulation, differentiation, and apoptosis [56]. Remarkably, pharmaceutical companies use diosgenin as a principal precursor compound for the manufacturing of several steroidal drugs [48]. Diosgenin is also an attractive molecule with multifaceted properties that has found application in pharmaceutical, functional food, and cosmetic industries. In this review we provide an in-depth evaluation of literature on diosgenin and its pharmacodynamics and pharmacokinetics, and discuss several of its novel derivatives and nanoformulations that increase its bioavailability and therapeutic efficacy. Diosgenin, over the years, has provided abundant data on the prevention and treatment of various inflammation-driven diseases, including cancers [36] (Figure 2).

## 2. Fenugreek Seed Bioactive Compounds

Fenugreek contains several chemical constituents, such as alkaloids, steroidal sapogenins, saponins, flavonoids, lipids, amino acids, and carbohydrates. Diosgenin is a major bioactive steroidal sapogenin in the fenugreek seed, which is reported to have chemopreventive and therapeutic effects against inflammation and chronic inflammation-driven cancers in preclinical in vitro and in vivo models of cancer [36,57]. Table 1 illustrates the major constituents of fenugreek seeds and leaves. 

## 3. In Vitro Anti-Cancer Effects of Diosgenin

**Figure 3:**
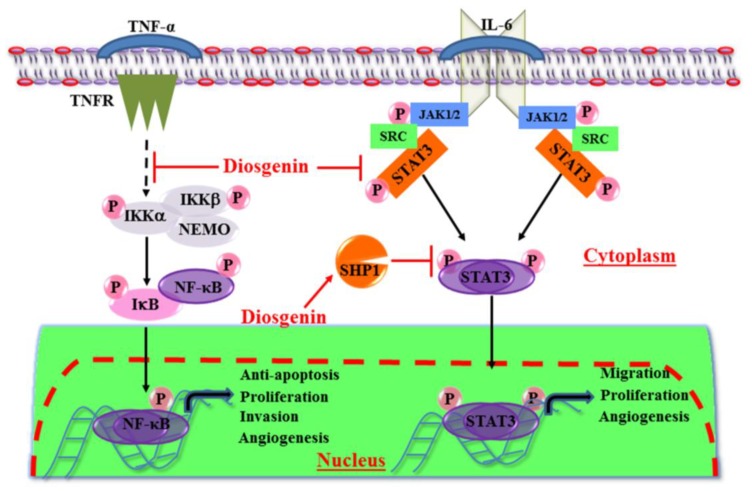
Role of diosgenin in NF-κB and STAT3 signaling pathways. Diosgenin abrogates TNF-α -induced activation of NF-κB and IL6-induced STAT3 signaling pathways in tumor cells. Diosgenin can hence prevent proliferation, invasion and angiogenesis; and induce apoptosis, a characteristic vastly looked for in cancer therapy.

Diosgenin, the major steroidal sapogenin in the fenugreek seed, has been shown to potently suppress constitutively-activated pro-inflammatory and pro-survival signaling pathways in a variety of cancer cells, and induced apoptosis [58]. Some of the earlier studies by Shishodia and Aggarwal [58] reported that diosgenin abrogated TNF-α-induced NF-κB activation and suppressed osteoclastogenesis in RAW 264.7 macrophage cells [58]. In Her-2 positive breast cancer cells, diosgenin inhibited the expression of AKT, mTOR, JNK and their associated pro-survival signaling pathways, and induced apoptosis in these cells [53]. In another study by Li et al., they reported that diosgenin could inactivate the STAT3 signaling pathway in hepatocellular carcinoma (HCC) cells, by inhibiting intracellular signaling molecules such as c-SRC, JAK1, and JAK2 (Figure 3). Diosgenin also suppressed STAT3 transcriptional activity and the expression of its downstream gene products involved in proliferation, invasion and metastasis. In addition, diosgenin sensitized HCC cells to doxorubicin and paclitaxel, and synergistically augmented apoptosis, thereby suggesting that diosgenin is a potential bioactive compound for the treatment of HCC and other cancers [54]. Diosgenin inhibited proliferation, AKT and JNK in a dose- and time-dependent manner and induced caspase-dependent apoptosis in A431 and Hep2 skin squamous cell carcinoma cells [59]. HT-29 colon cancer cells have been reported to be resistant to TRAIL-induced apoptosis. Diosgenin was shown to sensitize the HT-29 colon cancer cell to TRAIL. In addition, it potently suppressed cell proliferation and induced apoptosis by suppressing the p38/MAPK signaling pathway and the overexpression of DR5 [60]. Furthermore, Romero-Hernandez et al. [61] demonstrated that diosgenin-derived thio(seleno)ureas and glycomimetics, bearing a 1,2,3-triazolyl tether on C-3, showed more potent anti-cancer activity against MDA-MB-231 and MCF-7 breast cancer cells, HepG2 hepatocellular carcinoma cells, and induced apoptosis, compared to its parent compound diosgenin [61]. In another study, diosgenin conjugated to methotrexate was found to be more potent in inhibiting the growth of transport-resistant breast cancer cells and dihydrofolate reductase (enzyme involved in DNA synthesis), compared to the parent diosgenin [62]. In chronic myeloid leukemia cells, diosgenin-induced autophagy inhibited the mTOR signaling pathway and induced apoptotic cell death [63]. Diosgenin induced cytotoxicity and significantly inhibited the growth and proliferation of MCF-7 breast cancer cells in a dose- and time-dependent manner. Diosgenin was also shown to inhibit *N*-nitroso-*N*-methylurea-induced breast cancer in rats [64]. 

Of several factors that contribute to the sustained growth and proliferation of tumors, one important factor is the abundant neovascularization or formation of new micro blood vessels at the tumor site, or within the tumor. This process is also known as tumor angiogenesis. Thus, the formation of new blood vessels in tumors actively supplies the essential nutrients and growth factors that allow tumors to acquire the ability to reject chemotherapeutic drugs and develop chemoresistance [27,65,66,67,68]. Diosgenin is also a potent inhibitor of cancer cell invasion, migration, and tumor-associated angiogenesis [27,69]. He et al., reported that diosgenin inhibited the invasion and migration of triple-negative breast cancer cells and was associated with the concomitant suppression of actin polymerization, phosphorylation of Vav2, and activation of Cdc42 oncoprotein expression. These proteins have been shown to be involved in the initiation of cancer cells’ invasive and migratory potential [70]. Similarly, diosgenin was found to inhibit PC3 androgen-independent prostate cancer cell invasion and migration. The inhibitory effect was mediated by the downregulation of matrix metalloproteinase (MMP)-2 and MMP-9, the key enzymes in matrix degradation and stroma invasion. Furthermore, diosgenin also downregulated the tissue inhibitors of metalloproteinase (TIMP)-2, vascular endothelial growth factor (VEGF), extracellular regulated kinase (ERK), Janus kinase (JNK), phosphotidyl-inositol-3 kinase/protein kinase B (PI3K/AKT), and NF-κB transcriptional activity [56]. In addition, diosgenin was reported to inhibit the expression of E-cadherin, integrin 5a and 6b, invasion, migration, and angiogenesis in hypoxia-sensitive BGC-823 gastric cancer cells [71]. Diosgenin was reported to inhibit proliferation of ER-positive MCF-7 breast cancer cells by the upregulation of the p53 tumor suppressor gene and activation of caspase 3, while it downregulated BCL2 in ER negative MDA-MB-231 triple-negative breast cancer cells [72]. Diosgenin, either alone, or in combination with thymoquinone, inhibited A431 and Hep2 squamous cell carcinoma cell proliferation, increased the Bax/Bcl2 ratio, and induced caspase 3-mediated apoptosis [59] (Table 2). The potential effect of diosgenin on NF-κB and STAT3 signaling pathways in tumor cells, is shown in Figure 3.

## 4. In Vivo Anti-Cancer Effects of Diosgenin

In addition to in vitro inhibition of cancer cell proliferation by dietary fenugreek seeds and its bioactive constituent diosgenin, several studies have provided evidence that diosgenin is a potent inhibitor of tumor growth in vivo in rodent models of cancer. In a rat colorectal tumor model, administration of diosgenin, given during the promotional stage, reduced azoxymethane (AOM)-induced colonic aberrant crypt foci formation [79]. Similarly, Malisetty et al., showed that diosgenin, at a dose of 15 mg/kg, significantly suppressed both the incidence and invasive potential of AOM-induced rat colon adenocarcinoma mass by 60% and colon tumor multiplicity (adenocarcinomas/rat) by 68% [80]. However, in the murine model of AOM/dextran sodium sulfate-induced colon aberrant crypt foci, diosgenin at doses of 20, 100 and 200 mg/kg b.w. in the diet did not reduce adenocarcinoma mass; nonetheless, a significant reduction in tumor multiplicity was observed with all three doses tested [81]. In another study, diosgenin (at a dose of 10 mg/kg b.w. administered intra-tumorally) significantly inhibited the growth of MCF-7 and MDA-MB-231 human breast cancer xenografts in mice [72]. In another study using inbred T739 mice, diosgenin was shown to significantly inhibit the growth of mouse LA795 lung adenocarcinoma tumors by 33.94% [82]. Diosgenin, at a dose of 80 mg/kg administered by oral gavage, was reported to inhibit the growth of oral tumors in a DMBA-induced hamster buccal pouch model [64]. Diosgenin, in combination with thymoquinone, exhibited significant tumor growth inhibition in a mice xenograft model [59]. Therefore, diosgenin modulates multiple targets and suppresses tumor growth in preclinical models of cancer. However, diosgenin’s poor solubility in organic solvents and its lack of bioavailability greatly hinder its translational process as a therapeutic compound. Further clinical trials are required to evaluate its potential either as a preventive or therapeutic anti-cancer agent. 

## 5. Semisynthetic Derivatives of Diosgenin That Exhibit Anti-Cancer Activity

Diosgenin is used in the pharmaceutical industry as the main precursor in the synthesis of steroids [83]. It has the ability to penetrate cell membranes and bind to specific receptors [84]. Steroidal sapogenins are bioactive molecules that have shown exceptional antiproliferative activity against several human cancer cells. By making specific changes in the steroidal structure of diosgenin, it can affect its biological activity. In a recent report, using diosgenin as the parent molecule, the authors synthesized two novel steroidal oxime compounds that showed significant antiproliferative activity on cervical cancer cells and human lymphocytes. These compounds induced apoptosis and activated caspase 3 [85]. In another study Mohammad et al., reported on the anti-proliferative activity of diosgenin and its semi-synthetic derivatives against breast (HBL-100), colon (HCT-116 and HT-19) and lung (A549) cancer cells. A structure-activity relationship study revealed that the potent anti-proliferative activity was mainly attributed to the analogs with the simple phenyl R moiety or electron-withdrawing ortho-substituted R moieties attached to the parent diosgenin [86]. In another study, diosgenin was used as a parent compound to synthesize 1α-hydroxysolasodine; it showed significant anti-cancer activity against prostate cancer (PC3), cervical carcinoma (HeLa), and hepatocellular carcinoma (HepG2) cells [87]. Twelve different analogs of diosgenin containing a long chain fatty acid/ester of diosgenin-7-ketoxime exhibited anti-cancer activity when tested against a panel of cancer cell lines. Compound 16 in this series exhibited potent anti-proliferative activity against DU145 prostate cancer cells, which was associated to the suppression of lipopolysaccharide-induced activation of TNF-α and IL6. The compound was also identified as safe, with a maximum tolerated dose of 300 mg/kg in Swiss albino mice [88]. In a recent article by Ghosh et al., they reported the synthesis of diosgenin functionalized iron oxide nanoparticles that exhibited anti-breast cancer activity by inhibiting proliferation and migration, and by inducing apoptosis [89].

## 6. Conclusions and Future Perspectives

Compounds derived either from medicinal or dietary plant sources embrace distinct advantages, such as novel bioactive structures, low toxicity, and being multi-targeted in abrogating oncogenic processes; thereby, they may form the source of improved therapeutic options. A vast body of pre-clinical experimental evidence suggests that diosgenin has great potential as an anti-cancer agent. In this review we have compiled and analyzed the role of diosgenin in modulating various oncogenic transcription factors and intracellular molecular targets that drive tumor initiation, progression and metastasis. It is well known that the majority of cancers are a consequence of chronic inflammation, infection, dysfunctional cell death mechanisms, and deregulation of cell cycle molecules. The ability of diosgenin to prevent carcinogenesis by acting as an anti-oxidant and anti-inflammatory agent, and its ability to induce apoptosis of cancer cells, suggests that it can be useful as an anti-carcinogenic agent. Due to the complexities in the cellular processes involved, several new studies need to be conducted to decipher the exact molecular targets that can be exploited to prevent cancer progression. Interestingly, there are 12 reported clinical trials on fenugreek seed extract on a variety of human ailments, as reported in www.clinicaltrials.gov. However, to date, there are no cancer-related clinical trials reported either on diosgenin or on fenugreek seed extract. Several novel synthetic diosgenin derivatives have been shown to improve its anti-cancer efficacy. Several nano-formulations and delivery systems of diosgenin are also shown to improve its bioavailability. In conclusion, several challenges such as developing novel delivery systems, pharmaceutical formulations, and semi-synthetic derivatives that are water soluble, need to be overcome to uncover diosgenin’s benefits either as a chemopreventive or therapeutic agent.

## Figures and Tables

**Table 1:** Main phytochemical constituents of fenugreek (*T. foenum-graecum*).

Class of Compounds	Phytochemical Constituents	Reference
Steroidal sapogenins	Diosgenin, Yamogenin, Smilagenin, Sarsasapogenin, Tigogenin, Neotigogenin, Gitogenin, Yuccagenin, Saponaretin	[40]
Flavonoids	Quercetin, Rutin, Vitexin, Isovitexin	[40]
Saponins	Graecunins, Fenugrin B, Fenugreekine, Trigofoenosides A–G	[40]
Alkaloids	Trimethylamine, Neurin, Trigonelline, Choline, Gentianine, Carpaine, and Betain	[40]
Fibers	Gum, Neutral detergent fiber	[40]
Lipids	Lipids, Triacylglycerols, Diacylglycerols, Monoacylglycerols, Phosphatidylcholine, Phosphatidylethanolamine, Phosphatidylinositol, Free fatty acids	[40]
Others	Coumarin, Amino acids, Vitamins, Minerals. 28% Mucilage; 22% Proteins; 5% of a stronger swelling, Bitter fixed oil	[40]

**Table 2:** In vitro anti-cancer effects of diosgenin.

Cancer model	Cell Lines	Diosgenin Dose	Molecular Target	References
Breast carcinoma	Estrogen receptor positive and estrogen receptor negative human breast cancer MCF-7 and MDA 231 cells	20 μM and 30 μM	Inhibition of cell proliferation Induces apoptosis	[72]
	MDA-MB-231 breast cancer cells	20 μM, 40 μM, and 60 μM	Downregulation of Bcl2	[57]
	Her2 over-expressing breast cancer cells	5–20 μM	Modulation of Akt, mTOR, and JNK phosphorylation	[53]
	MCF-7 breast cancer cells	20 μM and 40 μM	Upregulation of p53 tumor suppressor gene	[73]
Hepatocellular carcinoma	C3A, HUH-7, and HepG2 cells	50 μM and 100 μM	Downregulation of STAT3 signaling pathway Upregulates SH-PTP2 expression Induces apoptosis Potentiates the apoptotic effects of doxorubicin and paclitaxel	[54]
Prostate carcinoma	PC3 cells	5 μM, 10 μM, and 20 μM	Downregulates NF-κB signaling pathway Inhibits matrix metalloproteinases Inhibits invasion and migration of cells	[56]
Osteosarcoma	1547 cells	40 μM, 80 μM, and 100 μM	Inhibits cell proliferation Induces apoptosis	[55]
	1547 cells	40 μM	Inhibits cell proliferation Induces apoptosis Upregulation of p53 tumor suppressor gene	[74]
Human erythroleukemia	HEL cells, K562 cells	40 μM	Inhibits NF-κB signaling pathway	[75]
	HEL cells	40 μM	Inhibits proliferation Induces apoptosis Upregulation of p21	[76]
Human Laryngocarcinoma Human Melanoma	HEp-2 cells M4Beu cells	40 μM	Inhibits cell proliferation Induces caspase-3 dependent apoptosis Upregulates p53 tumor suppressor gene	[77]
Human cancer cells	Human epithelial carcinoma cell line (A431), human NSCLC cell line (A549), human ovarian cancer cell line (A2780), Human erythroleukemia (K562) and Dukes’ type C, colorectal adenocarcinoma (HCT-15)	10 mmol/L	Induces apoptosis via mitochondrial dependent pathway	[78]
	Multiple myeloma (U266), leukemia (U937), and breast cancer (MCF-7)	50 μM and 100 μM	Inhibits NF-κB signaling pathway	[58]
